# Vascular dysfunction promotes regional hypoxia after bevacizumab therapy in recurrent glioblastoma patients

**DOI:** 10.1093/noajnl/vdaa157

**Published:** 2020-11-17

**Authors:** Elizabeth R Gerstner, Kyrre E Emblem, Yi-Fen Yen, Jorg Dietrich, Justin T Jordan, Ciprian Catana, Kevin Lou Wenchin, Jacob M Hooker, Dan G Duda, Bruce R Rosen, Jayashree Kalpathy-Cramer, Rakesh K Jain, Tracy T Batchelor

**Affiliations:** 1 Stephen E. and Catherine Pappas Center for Neuro-Oncology, Massachusetts General Hospital, Boston, Massachusetts, USA; 2 Department of Diagnostic Physics, Oslo University, Oslo, Norway; 3 Athinoula A. Martinos Center for Biomedical Imaging, Massachusetts General Hospital, Charlestown, Massachusetts, USA; 4 Edwin L. Steele Laboratories, Department of Radiation Oncology, Massachusetts General Hospital, Boston, Massachusetts, USA; 5 Department of Neurology, Brigham and Women’s Hospital, Boston, Massachusetts, USA; 6 Harvard Medical School, Boston, Massachusetts, USA

**Keywords:** antiangiogenic therapy, glioblastoma, hypoxia, vascular normalization

## Abstract

**Background:**

Hypoxia is a driver of treatment resistance in glioblastoma. Antiangiogenic agents may transiently normalize blood vessels and decrease hypoxia before excessive pruning of vessels increases hypoxia. The time window of normalization is dose and time dependent. We sought to determine how VEGF blockade with bevacizumab modulates tumor vasculature and the impact that those vascular changes have on hypoxia in recurrent glioblastoma patients.

**Methods:**

We measured tumor volume, vascular permeability (Ktrans), perfusion parameters (cerebral blood flow/volume, vessel caliber, and mean transit time), and regions of hypoxia in patients with recurrent glioblastoma before and after treatment with bevacizumab alone or with lomustine using [^18^F]FMISO PET-MRI. We also examined serial changes in plasma biomarkers of angiogenesis and inflammation.

**Results:**

Eleven patients were studied. The magnitude of global tumor hypoxia was variable across these 11 patients prior to treatment and it did not significantly change after bevacizumab. The hypoxic regions had an inefficient vasculature characterized by elevated cerebral blood flow/volume and increased vessel caliber. In a subset of patients, there were tumor subregions with decreased mean transit times and a decrease in hypoxia, suggesting heterogeneous improvement in vascular efficiency. Bevacizumab significantly changed known pharmacodynamic biomarkers such as plasma VEGF and PlGF.

**Conclusions:**

The vascular signature in hypoxic tumor regions indicates a disorganized vasculature which, in most tumors, does not significantly change after bevacizumab treatment. While some tumor regions showed improved vascular efficiency following treatment, bevacizumab did not globally alter hypoxia or normalize tumor vasculature in glioblastoma.

**Key Points**

- The degree of tumor hypoxia is variable in patients with recurrent glioblastoma.
- Inefficient vasculature with increased vessel caliber is associated with regional hypoxia.
- Bevacizumab (10 mg/kg) does not globally alter tumor hypoxia in recurrent GBM.

**Importance of the Study**

Hypoxia is a potent mediator of immunosuppression and treatment resistance. In this study, we characterized the vascular inefficiency associated with tumor hypoxia in patients with recurrent glioblastoma and found that there was increased cerebral blood flow/volume and increased vessel caliber within hypoxic regions. After bevacizumab treatment, this inefficient vasculature did not significantly change in the majority of the tumor volume, suggesting an intrinsic vascular resistance to bevacizumab. However, some subregions where hypoxia resolved showed improved vascular efficiency as evidenced by improved mean transit time. Advanced imaging can measure the impact of antiangiogenic therapy on vessel function and structure and, thus, should be explore as a noninvasive biomarker of response to help optimize therapy.

Hypoxia is a potent mediator of treatment resistance in cancer and has been associated with reduced survival in patients with glioblastoma (GBM).^1–3^ The abnormal tumor vasculature is a driver underlying tumor hypoxia and thus targeting abnormal vasculature with antiangiogenic therapy has been attempted in many cancer types, including GBM.^4^ In GBM, however, there has been no improvement in overall survival with bevacizumab or other antiangiogenic agents.^5–7^

Antiangiogenic therapy may reverse the negative impact of hypoxia by improving tumor oxygenation through improved perfusion but could also exacerbate hypoxia through excessive vascular pruning.^4^ Using various imaging methods to measure tumor vasculature and oxygenation status, GBM human patient-derived rat orthotopic xenograft models and human studies have provided insights into the complex interaction between vasculature, hypoxia, and tissue oxygen extraction fraction in the setting of bevacizumab treatment.^8,9^ Structural changes in vascular morphology may occur but do not necessarily translate into functional improvement in oxygenation.^10,11^ GBMs are heterogeneous tumors and prior studies have shown heterogeneous responses in perfusion and oxygenation within tumors and across patients.^8,12,13^ For example, in a phase II trial in newly diagnosed GBM patients, cediranib transiently increased perfusion for nearly 2 months, while in other patients, it did not change or actually went down. The former patients survived ~9 months longer than the latter.

As some patients benefit from antiangiogenic therapy, there is a need to better understand the structural and functional vascular mechanisms that control perfusion and tumor hypoxia to understand if bevacizumab can beneficially modulate tumor vasculature in some patients but not in others. Using [^18^F]FMISO PET, a PET tracer that is taken up by hypoxic cells, and dynamic susceptibility contrast (DSC) perfusion MRI, we sought to determine the level of vascular efficiency underlying hypoxic tissue in recurrent GBM patients and to assess whether bevacizumab modulates vascular efficiency to reverse or worsen tumor hypoxia on both a global tumor scale as well as in subset regions of the tumor.

## Materials and Methods

### Patient Population

Patients with recurrent GBM to be treated with the standard dose of bevacizumab 10 mg/kg every 2 weeks were eligible to participate in this study. Patients could receive concomitant chemotherapy with lomustine every 6 weeks as well. Both bevacizumab and lomustine are approved therapies for GBM and the combination has been shown to improve progression-free survival but not overall survival.^14^ Measurable disease, defined by at least one lesion that could be measured in at least one dimension (longest diameter to be recorded) as > 10 mm, was required. Patients with lower grade tumors that had progressed to GBM were eligible and all patients had to be >12 weeks from the completion of radiation. All patients signed informed consent, and this trial was approved by the Dana-Farber Harvard Cancer Center IRB (NCT02076152) in accordance with U.S. Common Rule. Patients underwent a baseline simultaneous PET-MRI scan (using the BrainPET prototype integrated with the 3T TimTrio MR scanner, Siemens Healthineers) prior to starting bevacizumab and prior to the second and third doses of bevacizumab (week 2 and week 4 of treatment). Additionally, MRI-only scans were performed 1 day after the first bevacizumab infusion, and then every 6 weeks in the lomustine cohort or every 8 weeks in the bevacizumab monotherapy cohort (Supplementary Figure 1).

### MRI Acquisition

T2-weighted sampling perfection with application-optimized contrasts using different flip-angle evolution (T2SPACE), fluid-attenuated inversion recovery (FLAIR), dynamic contrast enhanced (DCE), dynamic susceptibility enhanced (DSC), diffusion tensor imaging (DTI), pre- and postcontrast T1-weighted and magnetization-prepared rapid gradient-echo (MPRAGE) MR images (see Supplementary Data for parameter specifics) were acquired as previously described.^13^ A 1-channel transmit combined with either an 8- or 32-channel receive radiofrequency coil array built to minimize 511 keV photon attenuation were used for the study.^15^

### PET Acquisition

[^18^F]FMISO, a PET tracer that is taken up by viable hypoxic cells and is not influenced by perfusion, was produced on site or purchased from the Brigham and Women’s Hospital Biomedical Imaging Research Core.^16^ The radiotracer dose, 3.7 MBq/kg (0.1 mCi/kg with a maximum of 260 MBq, 7 mCi) was administered intravenously as a 30-s bolus shortly after the start of the PET data acquisition. PET data were acquired for 20 min 110 min post-radiotracer administration. PET images were reconstructed using the Ordinary Poisson Ordered Subset Expectation Maximization (OP-OSEM) 3D algorithm from prompt and random coincidences, normalization, attenuation (using an MR-based approach (cite 10.2967/jnumed.113.136341) and scatter coincidences sinograms using 16 subsets and 4 iterations. The reconstructed PET volume consisted of 153 slices with 256 × 256 pixels (1.25 × 1.25 × 1.25 mm^3^).

### MRI and PET Analysis

Apparent diffusion coefficient (ADC) maps were calculated from diffusion-weighted MRIs acquired with 5 b-values (0 up to 2200) and processed with in-house developed software written in MATLAB (MathWorks Inc). Gradient-echo and spin-echo DSC-MRI data were used to calculate macroscopic-vessel and microscopic-vessel cerebral blood volume (CBV) and cerebral blood flow (CBF) maps, respectively, using NordicICE (NordicNeuroLab AS).^17^ Vessel size index (VSI) and mean transit time (MTT) were also estimated from the DSC data.^17^ VSI quantifies the average vessel caliber in an image voxel.^18^ The DCE-MRI data were processed using in-house custom software written in MATLAB to obtain maps of Ktrans based on the 2-parameter Tofts model.^19^ Population-level arterial input functions (AIFs) were used for DCE analysis; DCE was used only to calculate Ktrans.

To assess median values within the tumor and peritumoral regions, all structural MRI sequences (FLAIR, MPRAGE pre/postcontrast), and parameter maps from DTI, DSC, and DCE were registered to the T2SPACE MR images using the BRAINSFit module in 3D Slicer. A deep-learning algorithm (DeepNeuro) was used to initially segment contrast enhancing tumor on the MPRAGE images (excluding regions of necrosis or blood) and abnormal FLAIR hyperintensity on the FLAIR images.^20^ These regions of interest (ROIs) were reviewed and edited as needed (by ERG). Median tumor values for ADC, Ktrans, macroscopic- and microscopic-vessel CBV and CBF, and VSI were calculated from the contrast enhancing ROI. Distinct tumors in individual patients were evaluated separately to assess if there were heterogeneous responses across tumors.

The PET images were analyzed as previously described.^21^ Regional hypoxic volume (HV) was determined by thresholding the ratio of the standardized uptake values (SUV) of [^18^F]FMISO in the brain to cerebellum above 1.2 also as previously described.^21^ Since some patients had multiple tumors, we identified the HV region within the union of each contrast enhancing ROI and its surrounding FLAIR ROI, so individual tumors could be evaluated separately. HV represents the magnitude of hypoxia within each tumor.

### Regional Hypoxic Tumor Analysis

To evaluate changes in tumor vasculature, the SUV maps where co-registered to the corresponding DSC-MRI before assessing values of macroscopic- and microscopic-vessel CBV/CBF and VSI, both within and outside the HV regions. Of particular interests were whether the tumor vasculature and HV regions changed longitudinally during bevacizumab treatment. To examine these potential longitudinal changes, all visits were registered to a common space (Montreal Neurological Institute space) to separate regions of changed and unchanged HV status within the entire brain. Four ROIs were identified: (1) tumor voxels that were never hypoxic before or during treatment; (2) tumor voxels that were hypoxic at baseline but no longer hypoxic by week 2 and week 4; (3) tumor voxels that were not hypoxic at baseline but became hypoxic by week 2 and week 4; and (4) tumor voxels that were hypoxic at baseline and remained hypoxic at week 2 and week 4 (Supplementary Figure 2).

### Blood Biomarkers

Serial blood monitoring was performed to assess circulating levels of plasma biomarkers of angiogenesis and inflammation. The blood was processed as previously described.^13^ Briefly, plasma samples were collected in EDTA-containing tubes, separated by centrifugation, aliquoted, and stored at −80°C until protein measurements were performed. The following plasma biomarkers were measured, VEGF, placental growth factor (PIGF), soluble (s)VEGFR-1, VEGF-C, VEGF-D, sTie2, and fibroblast growth factor (bFGF) using the Human Angiogenesis Panel Kit; interferon-gamma (IFN-γ), interleukin (IL)-1β, IL-6, IL-8, IL-10, and tumor necrosis factor (TNF)-α using the Human Proinflammatory Panel Kit by multiplexed array (Meso-Scale Discovery); and stromal cell-derived factor (SDF1)-α, sVEGFR2, and Ang-2 using single analyte ELISA kits.

### Statistics

Wilcoxon signed rank test was used to assess for differences in each imaging parameter or the blood biomarkers compared to the baseline visit. Except for the HV region analysis, if a patient had multifocal GBM, each tumor focus was compared separately to itself. For the HV region analysis, all visible tumor region was included in the analysis. Given the small sample size and exploratory nature of the study, analyses that would require corrections for multiple testing were not performed.

## Results

### Patients

**Figure 1.**
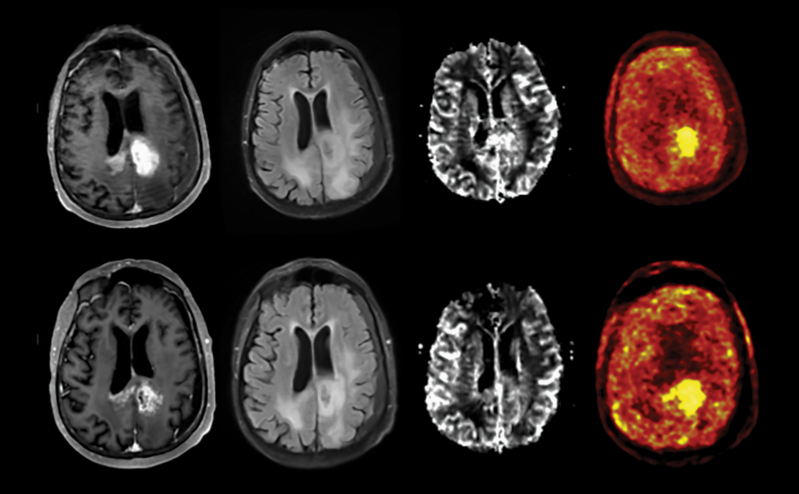
Example from one recurrent glioblastoma patient (FMS_01) demonstrating longitudinal change from baseline (top row) to week 3 (bottom row) with bevacizumab in contrast enhancement (A), T2/FLAIR (B), macroscopic CBV (C), and SUV map (D).

Eleven recurrent GBM patients were enrolled; 9 received lomustine with bevacizumab and 2 received bevacizumab alone (Supplementary Table 1). Treatment was well tolerated with no unexpected side effects, and there were no adverse events related to the [^18^F]FMISO-PET-MRI scans. All patients progressed on treatment and only 1 patient with an IDH1-mutant tumor remained alive at last follow-up (34 months). Similar to prior studies with bevacizumab, median progression-free survival was 4.0 months and median overall survival was 7.4 months in the entire cohort. Because of production challenges with [^18^F]FMISO, only 4 patients underwent all 3 FMISO-PET scans (all treated with both bevacizumab and lomustine). The remaining 7 patients had MRI scans alone at those visits. Figure 1 shows an example image from a representative patient.

### Vascular Changes in Response to Bevacizumab

When compared with baseline, there was a significant decrease in contrast enhancing disease volume, median tumor ADC, and median tumor Ktrans at day 1, week 2, and week 4 of treatment (Table 1, Supplementary Figure 3). Median tumor Ktrans remained significantly decreased at week 6. The volume of abnormal FLAIR hyperintensity significantly decreased at day 1 (*P* = .004) and week 2 (*P* = .02). Microscopic CBV significantly decreased at day 1 (Table 1). There were no other statistically significant changes in whole tumor measures of perfusion as reflected in median tumor CBF/CBV values for the entire cohort. In a subset of these patients, however, and, similar to prior reports, in 3 of 11 patients (27%) whole tumor microscopic CBF increased over 2 consecutive scans during bevacizumab. This finding suggests heterogeneity in tumor vascular response, with some patients showing increased perfusion during antiangiogenic treatment, whereas others do not.^12,13^

**Table 1.** MRI Parameters Within the Contrast Enhancing Tumor for All 11 Patients

Variables	Baseline	Day 1	Week 2	Week 4	Week 6
Volume (cc)	1666 (1048–2014)	1542 (831–1680) *N* = 21 *P* < .001	1645 (967–1900) *N* = 22 *P* < .001	1472 (927–1768) *N* = 20 *P* < .001	1579 (1373–1749) *N* = 21 NS
Median GE CBV	1.03 (0.84–1.24)	0.956 (0.57–1.29 *N* = 21 NS	1.22 (1.01–1.43) *N* = 22 NS	1.11 (0.73–1.55) *N* = 20 NS	1.15 (0.59–1.53) *N* = 21 NS
Median GE CBF	1.00 (0.83–1.36)	1.01 (0.64–1.20) *N* = 21 NS	1.15 (0.92–1.28) *N* = 22 NS	1.01 (0.76–1.48) *N* = 20 NS	1.08 (0.61–1.45) *N* = 21 NS
Median SE CBV	0.68 (0.51–0.84)	0.59 (0.46–0.77) *N* = 21 *P* = .01	0.67 (0.51–0.93) *N* = 22 NS	0.70 (0.55–0.86) *N* = 20 NS	0.58 (0.47–0.82) *N* = 21 NS
Median SE CBF	0.75 (0.55–0.90)	0.70 (0.57–0.86) *N* = 21 NS	0.77 (0.65–0.93) *N* = 22 NS	0.75 (0.67–1.01) *N* = 20 NS	0.74 (0.63–1.00) *N* = 21 NS
Median ADC	0.93 (0.77–1.02 × 10^3^)	0.00083 (0.73–0.98 × 10^3^) *N* = 21 *P* = .02	0.84 (0.76–0.93 × 10^3^) *N* = 19 *P* = .01	0.77 (0.63–0.83 × 10^3^) *N* = 20 *P* = .003	0.77 (0.68–0.82 × 10^3^) *N* = 21 *P* = .001
Median K^trans^	0.14 (0.06–0.26)	0.05 (0.03–0.09) *N* = 19 *P* < .001	0.08 (0.03–0.13) *N* = 18 *P* < .001	0.05 (0.02–0.14) *N* = 19 *P* < .001	0.06 (0.02–0.10) *N* = 19 *P* < .001

If a patient had multiple tumors, each tumor was evaluated separately so N represents the number of tumors available for analysis at each time point. Data are shown as median values and interquartile range.

ADC, apparent diffusion coefficient; CBF, cerebral blood flow; CBV, cerebral blood volume.

*P value from Wilcoxon test compared to baseline; Not adjusted for multiple testing. *N* = number of tumor pairs compared.

### Tumor Hypoxia and Associated Vascular Changes

**Figure 2.**
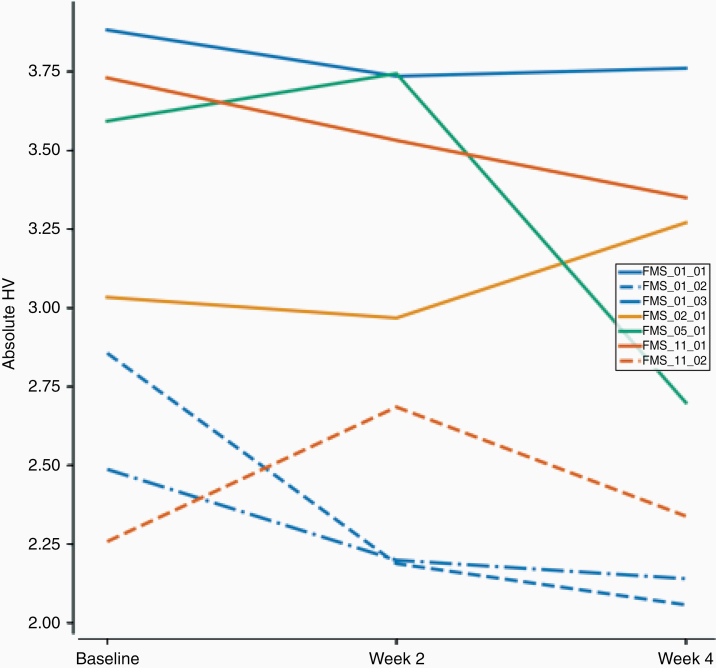
Longitudinal change in individual whole tumor hypoxic volume demonstrating variability in baseline magnitude of tumor hypoxia and limited change in hypoxia during bevacizumab treatment. Each line represents a single tumor. Patients with multiple tumors are shown in the same color. Only 2 tumors had a decrease in tumor hypoxia. All patients were treated with both lomustine and bevacizumab.

At baseline, the degree of tumor hypoxia as measured by HV was variable across the 11 recurrent GBM patients, including within individual tumors within the same patient (Figure 2). The median size of the HV region was 45% (range 40%–74%) of the total tumor area at baseline, 62% (43%–88%) at week 2, and 53% (20%–86%) at week 4. The change in individual tumor HV was variable with only 2 tumors showing a decrease in HV by week 4 and the others stable or increased (Figure 2). There was no significant longitudinal change in HV following bevacizumab.

**Figure 3.**
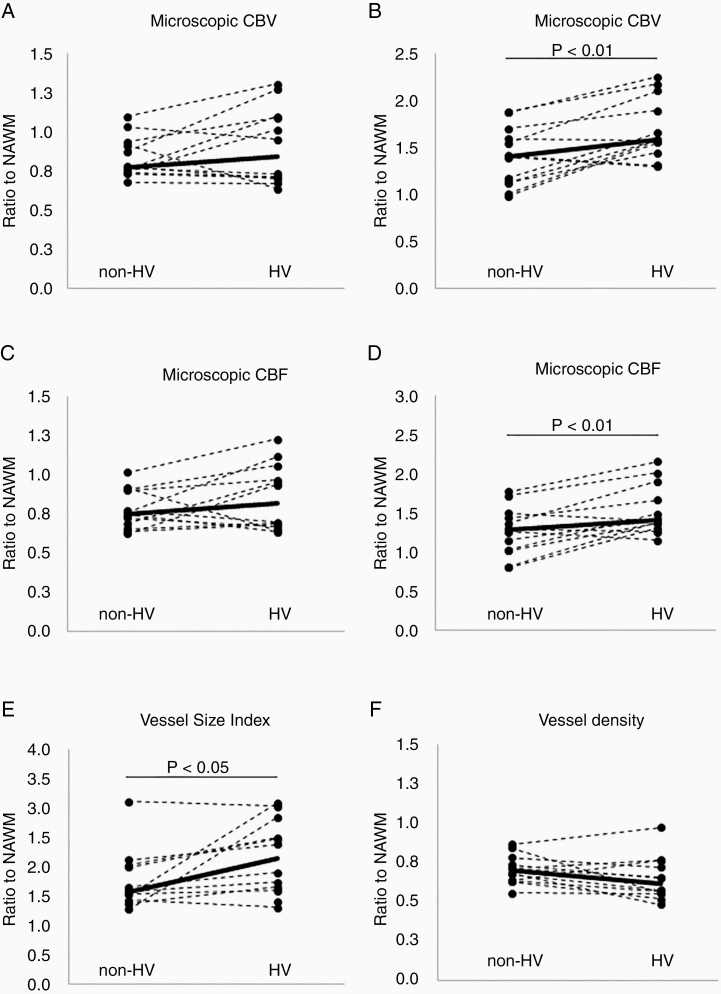
Changes in vascular parameters within hypoxic and nonhypoxic regions from each PET-MRI exam. Hypoxic regions demonstrate increased macroscopic CBV/CBF and vessel caliber compared to nonhypoxic regions, suggesting an inefficient vasculature within hypoxic regions. Owing to the paired analysis, data from all PET exams are included in the analysis (4 patients with 3 visits each). *P* value from Wilcoxon signed rank test. NAWM, normal-appearing white matter.

After combining the 12 visits in the 4 patients with [^18^F]FMISO-PET scans, panvascular macroscopic-vessel CBF was elevated within the HV tumor regions (median 1.41 vs 1.28 in the non-HV tumor regions, *P* = .01; Figure 3). Similar results were observed in the panvascular macroscopic-vessel CBV (median 1.58 in HV tumor regions vs 1.40 in the non-HV tumor regions, *P* = .01). The corresponding CBF and CBV levels in the microscopic-vessels were similar in the HV and non-HV regions. Consequently, VSI was elevated in the hypoxic tumor regions compared to nonhypoxic tumor regions (median 2.14 vs 1.57, respectively, *P* = .05).

### Longitudinal Changes in Tumor Vasculature and Hypoxic Regions

Following initiation of bevacizumab, the median size of the tumor region where hypoxia resolved was 12% (range 3%–18%) of the total tumor area, whereas the median size of regions where hypoxia developed was 2% (1%–4%). The ratio of hypoxic to nonhypoxic regions increased over time because of a relative decrease in the nonhypoxic region more than an increase in the hypoxic region, suggesting that the hypoxic region was changing less in response to bevacizumab (Supplementary Figure 4).

**Figure 4.**
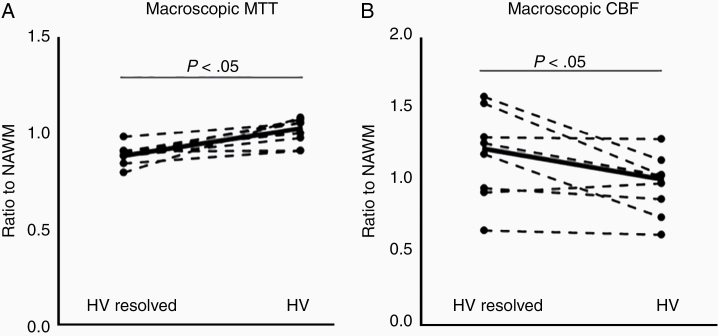
Differences in mean transit time (MTT) (A) and microscopic cerebral blood flow (CBF) (B) following treatment with bevacizumab in hypoxic regions (HV) and in regions where hypoxia resolved (HV resolved compared to the baseline exam. *P* value from Wilcoxon signed rank test. NAWM, normal-appearing white matter.

The total macroscopic MTT decreased to 88% of baseline levels in the tumor region where hypoxia resolved compared to the abnormal MTT in the region where hypoxia persisted (Figure 4). Since elevated MTT is associated with hypoxia, this decrease in MTT corroborates the importance of restoring normal vascular structure and function to relieving hypoxia.^22^ In the tumor regions where hypoxia developed, the total microscopic CBF was further elevated compared to the region where hypoxia persisted (Figure 4). There were no other statistically significant associations.

Longitudinal exploratory analysis revealed that tumor regions where hypoxia developed at week 2 and week 4 of treatment already had elevated CBF and VSI at baseline (Supplementary Figure 5). Regions that were never hypoxic or where hypoxia resolved with bevacizumab, maintained low CBF and VSI throughout. Regions that remained hypoxic throughout treatment, maintained elevated CBF and VSI.

### Blood Biomarkers

Serial blood samples were available from 9 patients. We observed a drop in plasma Ang2 (at day 1) and free VEGF (at day 1 and week 2) and an increase in PlGF (at day 1, and weeks 2 and 4) and sTie2 (at week 2) (Supplementary Table 2). No other significant differences were found in other circulating markers of angiogenesis or inflammation.

## Discussion

As expected, bevacizumab had a significant impact on tumor vascularity, specifically on vascular permeability, as measured by decreased contrast enhancement, decreased FLAIR hyperintensity, decreased ADC, and decreased Ktrans.^23^ In addition, consistent with published reports, bevacizumab changed known pharmacodynamic biomarkers such as PlGF, VEGF, and Ang2.^13^ Thus, bevacizumab treatment showed vascular biological effects in recurrent GBM patients. Since most patients also received lomustine, we could not separate the impact of lomustine, if any, on the vasculature. However, lomustine is not known to target endothelial cells, so was unlikely to significantly contribute to any vascular remodeling.^24,25^

Prior to treatment, the volume of tumor hypoxia as identified by increased [^18^F]FMISO uptake was variable with some tumors more hypoxic than others. Longitudinal changes in the tumor volume of hypoxic regions during bevacizumab treatment were variable, but most tumors did not experience significant change in the magnitude of hypoxia. Notably, the volume of tumor hypoxic regions decreased less than the nonhypoxic regions, suggesting greater resistance to bevacizumab therapy in areas that were already hypoxic. The lack of an increase in hypoxia could also be due to the fact that our population had recurrent GBM, where hypoxia is well established; so, further increase could not be detected.

In particular, hypoxic regions had the most abnormal vascular signature with elevated CBF, CBV and vessel caliber. Since there was no corresponding change in vessel density, these findings confirm that hypoxia is associated with an inefficient vasculature—there were more vessels of larger caliber underlying the increase in CBV and CBF. Larger vessels suggest that there are relatively fewer capillaries and, since capillaries are more efficient in delivering oxygen, the results suggest less efficient tumor vasculature and increased regional hypoxia.^26,27^ Mouse models in GBM support a shift from smaller capillaries to larger vessel calibers and our observations in humans confirm this increase in vessel caliber within tumors as a driving force behind regional hypoxia.^28^

Tumor hypoxia resulting from disorganized vascular structural changes leads to treatment resistance via several proposed mechanisms including an increase in immunosuppression, genomic instability, and a switch to anaerobic metabolism.^2^ Our results support the intrinsic treatment resistance of hypoxic regions as these regions of hypoxia persisted during treatment with bevacizumab at the standard 10 mg/kg dose every 2 weeks and the underlying vasculature remained abnormal. Antiangiogenic therapy, meant to target this abnormal vasculature, has complex effects, and we have yet to optimize its use as the dose may be critical for optimal efficacy, particularly when combined with other therapies.^29–31^ Prior studies have confirmed the significant impact antiangiogenic therapy can have on concomitant therapy—an impact that can be beneficial through improved tumor oxygenation—or detrimental through decreased chemotherapy penetration or worsening hypoxia.^12,13,32–34^

This persistence in hypoxia and abnormal vascular signature during bevacizumab treatment suggests that, at the conventional dose in GBM patients, bevacizumab is not reorganizing tumor vasculature to a more efficient overall state and, thus, not reversing the hostile hypoxic tumor microenvironment. There was intrapatient and intratumoral heterogeneity in the response to bevacizumab, however, as the tumor subregions that demonstrated a decrease in hypoxia during treatment also had an improvement in the vascular signature towards more efficient and shorter mean transit times (MTT). Similar improvements in MTT have been observed in GBM animal models treated with antiangiogenic therapy with a vascular normalizing response and in stroke where reperfusion is associated with improved MTT.^22,35^ Also, similar to prior studies, a subset of patients (27%) experienced an increase in microscopic perfusion, which has also been associated with normalization and improvement in oxygenation.^12,13^

Although the number of patients in our study was small and not all patients had PET scans (limiting the statistical analysis), the results highlight the critical impact of structural changes in tumor vasculature that lead to hypoxia and its downstream detrimental effects—even within 1 month of starting treatment. The impact that antiangiogenic therapy has on tumor vasculature needs to be optimized and alternative dosing schedules that enhance vascular normalization and improve tumor oxygenation globally may improve outcomes, particularly in the setting of concomitant therapy. Patient response may also be variable to vascular targeting therapy, highlighting the need to optimize noninvasive imaging to longitudinally monitor tumor response and potentially customize antiangiogenic therapies. Finally, development of other strategies—beyond solely targeting VEGF—to normalize tumor vessels would benefit from using the noninvasive imaging approaches described in this report.^34^

## Supplementary Material

vdaa157_suppl_Supplementary_Figures

vdaa157_suppl_Supplementary_Materials

## Notes

**Conflict of Interest:** J.T.J. reports consulting for CereXis pharmaceuticals, Recursion Pharmaceuticals, Health2047, Inc., and Navio Theragnostics; Royalties from Elsevier; D.G.D. received consultant fees from Bayer, Simcere and BMS and research grants from Bayer, Exelixis, and BMS. K.E.E.: Intellectual property rights (NordicNeuroLab AS, Bergen, Norway). J.K.C. reports research funding from GE and Genentech Foundation. R.K.J. received honorarium from Amgen and consultant fees from Chugai, Ophthotech, Merck, SPARC, SynDevRx and XTuit. R.K.J. owns equity in XTuit, Enlight, SPARC, and SynDevRx and serves on the Boards of Trustees of Tekla Healthcare Investors, Tekla Life Sciences Investors, Tekla Healthcare Opportunities Fund, and Tekla World Healthcare Fund. He is a member of the scientific advisory board of Accurius Therapeutics. No reagent or funding from any of these companies was used in the project. T.T.B. reports compensation from Genomicare scientific advisory board and compensation from Amgen as consultant, legal case.

## Notes

**Authorship Statement:** Experimental design and implementation: E.R.G., K.E.E., Y.F.-Y., J.D., J.T.J., C.C., J.H., D.G.D., B.R., J.K.-C., R.K.J., T.T.B.; analysis and interpretation of the data: all authors; all authors wrote the manuscript and have read and approved the final version.
