# Supplementary material for: Vascular dysfunction promotes regional hypoxia after bevacizumab therapy in recurrent glioblastoma patients
Source: Neurooncol Adv. 2020 Nov 17;2(1):vdaa157. doi: 10.1093/noajnl/vdaa157 (PMC7764510; doi:10.1093/noajnl/vdaa157)

**Supplementary Figures:**

**Supplementary Figure 1: Trial schema.** (A) Bevacizumab alone cohort (N=2); (B) Bevacizumab with lomustine cohort (N=9)**.**


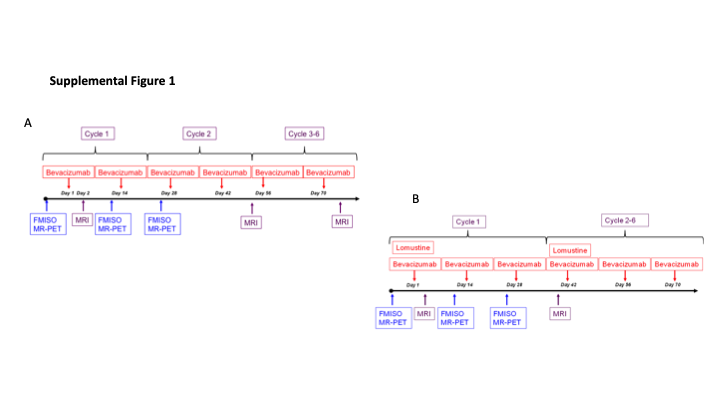


**Supplementary Figure 2:** Workflow for registering longitudinal scans to Montreal Neurological Institute (MNI) space so longitudinal changes in tissue oxygenation can be determined. HV (hypoxic volume) = region of hypoxia as determined by the [^18^F]FMISO PET scan (FMS_05).


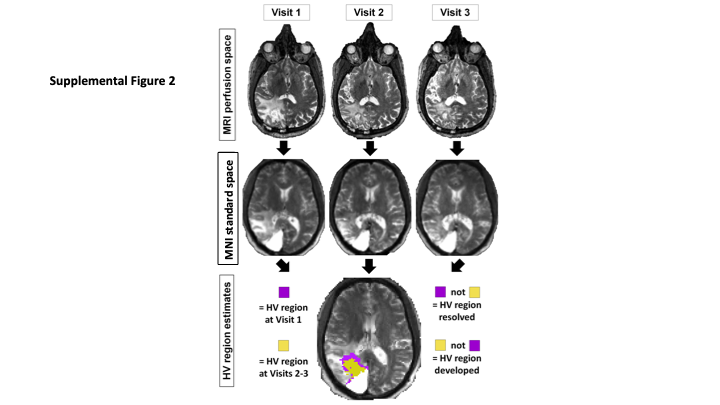


**Supplementary Figure 3:** Longitudinal change in MRI parameters.(A) percent change in contrast enhancement from baseline, (B) median tumor CBV, (C) median tumor Ktrans, and (D) median tumor ADC for all patients. If a patient had multiple tumors, each tumor was evaluated separately. All sequences were registered to 1mm isotropic space and the median tumor values for CBV, Ktrans, and ADC were calculated from the contrast enhancing region. The X axis is the visit time point and n=number of tumors available for analysis at that time point.


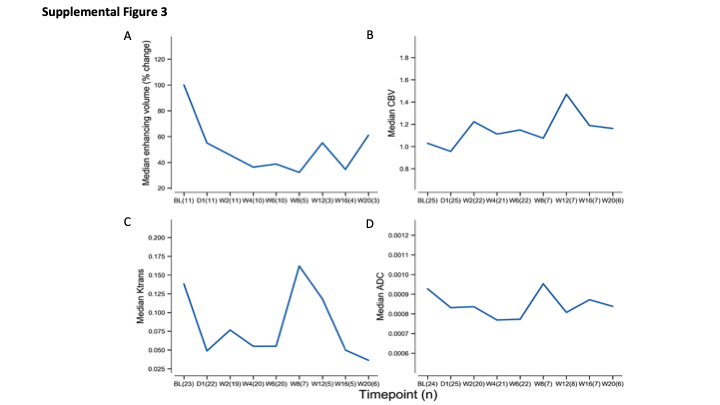


**Supplementary Figure 4:** Longitudinal change in the size of hypoxic volume (HV) and nonhypoxic volume (non-HV) regions of interest (A) and ratio of HV to non-HV tumor regions demonstrating less increase in the hypoxic regions with time (week 4 of bevacizumab) (B). HV was defined as ratio of the standardized uptake values (SUV) of [^18^F]FMISO in the brain to cerebellum above 1.2. [cc]; cubic centimeter


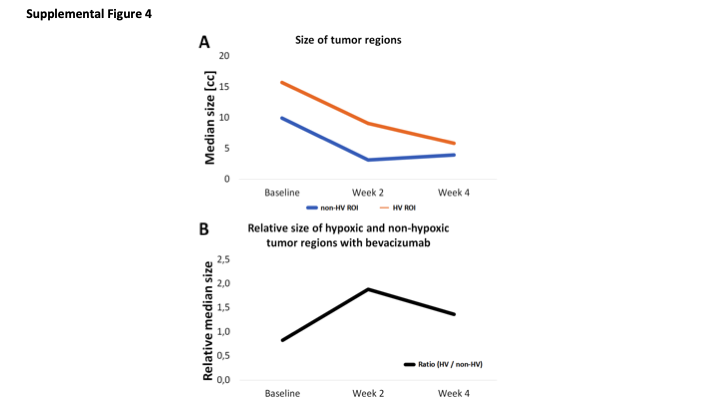


**Supplementary Figure 5:** Longitudinal vascular changes within different tumor regions. (A) Macroscopic cerebral blood volume (CBV); (B) Macroscopic cerebral blood flow (CBF); (C) Microscopic CBV (D); Vessel size index. Blue: tumor regions that were never hypoxic before or after bevacizumab treatment (non-HV). Orange: tumor regions that were hypoxic at baseline but no longer hypoxic by week 2 and week 4 (HV resolved). Gray: tumor regions that were not hypoxic at baseline but became hypoxic by week 2 and week 4 (HV developed). Yellow: tumor regions that were hypoxic at baseline and remained hypoxic at week 2 and week 4 (HV all). Regions that developed hypoxia or remain hypoxic have elevated perfusion and vessel caliber highlighting the abnormal vasculature in regions at risk of developing hypoxia or already are hypoxic. Median values with quartile ranges (Q1, Q3) shown for each parameter. NAWM; normal-appearing white matter.


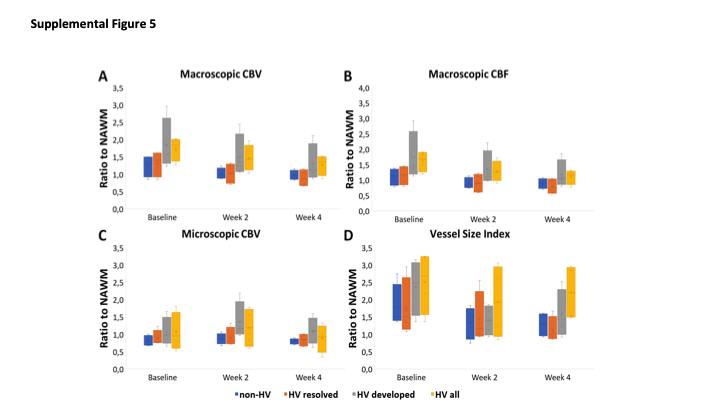

Supplement: vdaa157_suppl_Supplementary_Figures [file vdaa157_suppl_supplementary_figures.docx]
