# Supplementary material for: Vascular dysfunction promotes regional hypoxia after bevacizumab therapy in recurrent glioblastoma patients
Source: Neurooncol Adv. 2020 Nov 17;2(1):vdaa157. doi: 10.1093/noajnl/vdaa157 (PMC7764510; doi:10.1093/noajnl/vdaa157)
Supplement: vdaa157_suppl_Supplementary_Materials [file vdaa157_suppl_supplementary_materials.docx]

**Supplementary Information**

*MRI Acquisition Protocol*

Multi-slice 2D axial FLAIR images were acquired with TR 10000 ms, TE 73 ms, TI 2500 ms, slice thickness 5 mm, slice gap 1 mm, 25 slices, field of view (FOV) 256 mm X 224 mm, and matrix size 256 X 168. T1-pre and T1-post contrast 2D axial images were obtained with TR 250 ms, TE 4.58 ms, FA 90°, slice thickness 5 mm, slice gap 1 mm, 25 slices, FOV 256 mm X 224 mm, and matrix size 256 X 224. T2SPACE 3D images were acquired with TR 3200 ms, TE 404 ms, echo train length 141 with variable flip angles, slice thickness 1 mm, 192 slices, FOV 256 mm X 256 mm, and matrix size 256 X 256. MPRAGE 3D images were obtained with TR 2530 ms, 4 echoes with maximum TE 7.22 ms, slice thickness 1 mm, 176 slices, FOV 256 mm X 256 mm, and matrix size 256 X 256.

T1 mapping was performed by repeating a T1-weighted 3D gradient-echo acquisition six times, each at a different flip angle (FA). The imaging parameters were TR 6.3 ms, TE 2.51 ms, slice thickness 2.11 mm, 20 slices, FOV 256 mm X 208 mm, matrix size 128 X 71, acceleration factor of 3 and 4 averages. The six FAs were 2°, 5°, 10°, 15°, 20°, and 30°. Following the T1 mapping, DCE-MRI was acquired by using a similar protocol as T1 mapping, except for 10° FA and 1 average, and 250 measurements (frames) collected in 412 s, corresponding to a temporal resolution of 1.65 s. A bolus of 0.1 mmol/kg of Gd-DTPA (Magnevist^®^, Bayer AG, Germany) was injected 1 min after the DCE scan started, followed by 20 mL saline flush at 3.5 mL/s injection rate.

DSC-MRI was acquired by using a 2D echo-planer sequence with TR 1500 ms, TE 31 ms, FA 90°, slice thickness 5 mm, slice gap 1.5 mm, 11 slices, FOV 220 mm X 220 mm, and matrix size 128 X 128. A total of 99 frames were collected in 161 s, corresponding to a temporal resolution of 1.63 s. A bolus of 0.1 mmol/kg of Gd-DTPA was injected 30 seconds after the DSC scan started, followed by 20 mL saline flush at 3.5 mL/s injection rate. The Gd-DTPA injection for DCE-MRI served as preload for the DSC-MRI.

DCE-MRI and DSC-MRI data were motion corrected to align all volumes to the first volume of each dynamic series by using FSL.(28) Permeability transfer constant (Ktrans) maps were generated from motion corrected DCE-MRI data by using custom software in MATLAB based on Tofts Model with a constant T1 of 1000 ms and population-based AIF.(16,29) Maps of relative cerebral blood flow (rCBF) to normal apparent white matter (NAWM) tissue was obtained by using NordicICE (NordicNeuroLab AS, Bergen, Norway) with T1 leakage correction, population-based AIF, and automatic classification of NAGW areas based on cluster analysis.(17,30)

*PET Acquisition and Analysis*

Emission data were stored in list-mode format and re-binned into sinogram space using nearest neighbor approximation and axial compression (span 9 and maximum ring difference 67). The calculation of random coincidences was performed by sorting the delayed coincidences into delayed single maps, from which the total single rate as well as the variance reduced randoms were estimated.(31) The sensitivity data were acquired with a plane source scanned in 16 positions (with a 22.5**°** angular step), four hours per position. The normalization of sinogram was obtained by sorting the sensitivity data in the sinogram space.

Head attenuation map was obtained using an atlas-based MR-based attenuation correction method.(32) The evaluation of this method in various patient populations, including glioblastoma patients, revealed a mean absolute difference of 2.44% in the brain when compared to the gold standard, scaled CT. The scatter sinogram was obtained using a calculated method based on the single scatter estimation method.(33)
